# Epileptic seizure first aid practices of publics in Northwest Ethiopia 2021: Unsafe practices of nearly three-fourths of the community

**DOI:** 10.3389/fneur.2022.1032479

**Published:** 2022-11-15

**Authors:** Sintayehu Asnakew, Getasew Legas, Amsalu Belete, Getnet Mihretie Beyene, Assasahegn Tedla, Kirubel Shiferaw, Birhanu Mengist, Wubet Alebachew Bayih, Dejen Getaneh Feleke, Binyam Minuye Birhane, Ermias Sisay Chanie, Zelalem Birhan, Mengesha Birkie, Getachew Yideg Yitbarek

**Affiliations:** ^1^Department of Psychiatry, School of Medicine, College of Health Science, Debre Tabor University, Debre Tabor, Ethiopia; ^2^Department of Pediatrics and Child Health Nursing, College of Health Science, Debre Tabor University, Debre Tabor, Ethiopia; ^3^Department of Psychiatry, College of Medicine and Health Science, Wollo University, Dessie, Ethiopia; ^4^Department of Psychiatry, College of Medicine and Health Science, Injibara University, Injibara, Ethiopia; ^5^Departments of Biomedical Sciences, College of Health Science, Debre Tabor University, Debre Tabor, Ethiopia

**Keywords:** seizure, epilepsy, unsafe practice, south Gondar zone, community

## Abstract

**Background:**

Religious and sociocultural beliefs influence how people with epilepsy (PWE) are treated and cared for. Many communities in Africa and other developing countries, including Ethiopia, believe that epilepsy is caused by evil spirits and should be treated with herbal plants by traditional doctors and religious leaders. The combination of these sociocultural beliefs and the level of community awareness of epilepsy affect first aid practices in the management of epileptic seizures.

**Objective:**

This study aimed to assess epileptic seizure first aid practice of public and its associated factors in Northwest Ethiopia, south Gondar zone, Amhara, Ethiopia 2021.

**Methods:**

A community-based cross-sectional study was conducted using a previously adapted standard questionnaire. A multistage cluster sampling technique was applied. A total of 756 participants were approached and 741 respondents completed the questionnaire with a response rate of 98.02%. Data were entered into Epi data version 4.4.2 and then exported to Statistical Package for Social Science (SPSS) version 24 for analysis. Descriptive and analytical statistical procedures and bivariable and multivariable binary logistic regressions with odds ratios and 95% confidence interval (CI) were employed. The level of significance of the association was determined at *p* < 0.05.

**Results:**

Overall, 71.7% (95%CI: 68.3, 74.9) of the south Gondar community had unsafe practice measures in managing patients with seizure episodes. Individuals who were illiterate [adjusted odd ratio (AOR) = 1.99, 95%CI: 1.00.3.97] and participants who did not take training related to epilepsy (AOR = 2.07, 95%CI: 1.35, 3.17) and had poor knowledge about (AOR = 1.51, 95%CI: 1.06, 2.14) and a negative attitude toward epilepsy (AOR = 2.20, 95%CI: 1.50, 3.22) had unsafe practices compared to their counterparts. Conversely, participants who reached secondary education had safe practice measures (AOR = 0.4, 95%CI: 0.26, 0.63) in the management of epileptic seizures.

**Conclusions:**

In this study, large numbers of the south Gondar community had unsafe practice measures in managing people with epileptic seizure episodes. Greater emphasis should be laid on individuals who were illiterate, in the provision of health education and/or training for the community to help them to acquire good knowledge about epilepsy and develop a positive attitude toward epilepsy.

## Introduction

An epileptic seizure is defined as a brief episode of signs and/or symptoms associated with abnormally excessive or synchronized brain neuron activity (1). Two or more spontaneous seizures occurring more than 24 h apart are considered clinically indicative of epilepsy (2). The need for care and treatment of people with epilepsy (PWE) are influenced by religious and cultural beliefs (3).

Epilepsy affects at least 50 million people globally (4), with the majority living in developing countries (5). The burden of epilepsy in low-income countries is double compared to developed countries (6). In a study conducted in some African countries (Kenya, Tanzania, Uganda, Ghana, and South Africa), the prevalence of epilepsy was between 7 and 15 per 1,000 people (7), 0.9/1,000 in Sudan (8), and 6.5/1,000 in Saudi Arabia (9). Similarly, the incidence of epilepsy in Qatar was 174 per 100,000 people (10). Likewise, the adjusted prevalence estimates for lifetime and active epilepsy in a study conducted in rural Kenya were 41/1,000 and 11/1,000, respectively (11). Moreover, in the Zay society of Ethiopia, the prevalence rates of epilepsy are found to be 29.5/1,000 (12).

People with epilepsy are highly stigmatized in most parts of the world (13, 14) and have different levels of awareness, attitudes, and first aid practice measures regarding epilepsy in various cultures. Nevertheless, PWE generally encounter social stigma, there are public misconceptions about the treatment and first aid measures at the time of seizure attacks (15). Even though the psychosocial and economic impacts of epilepsy are considered in high-income countries, the treatment gaps in low-income countries remain large, ranging from 60 to 98% (16). In Ethiopia, more than 85% of PWE are untreated. Only 4% of the untreated cited cost as justification for not receiving treatment, and 90% were unaware of the availability of treatment (17). Epilepsy remains a major public health problem, not only because of its health implications, but also because of its social, cultural, psychological, and economic effects in developing countries (18, 19). PWE also experience physical, intellectual, psychological, and social limitations (20). In severe cases, it results in disability and death due to burns, drowning, and even depression-induced suicide (21). This is usually associated with public misbeliefs (22) and inadequate public knowledge and a negative attitude about epilepsy, myths, and misconceptions (23–25).

A cross-sectional study of people living with epilepsy in sub-Saharan Africa, India, and Zimbabwe showed that PWE had limited opportunities in education, employment, and healthcare compared to the other population without epilepsy (26–28). Research conducted in Karachi, Pakistan, and Southeastern Nigeria on the practices of school teachers and secondary school students toward children with epilepsy showed persistent unsafe practices toward the management of epileptic seizures (29, 30). Surprisingly, there is an unsafe practice of epilepsy even among medical students as indicated by a study conducted in Southern Nigeria (31). Similarly, a study by World Health Organization (WHO) on epilepsy first aid management practice of the population in suburban Senegal revealed that nearly 23.4% of PWE had unsafe treatment practices (32). According to the result of previous studies, the magnitude of unsafe practices ranges from 6 to 68.1% (33–36).

First aid is crucial to prevent people from getting harmed during seizure episodes. Lack of knowledge and misunderstandings about first aid measures related to epileptic seizures cause the chance of not taking effective measures or taking dangerous measures while managing a seizure episode (37).

Factors that affect first aid practices in the community in the management of epileptic seizures include sociodemographic variables (age, sex, residence, marital status, educational status, and occupational status), the presence of patients with epilepsy in a family, the presence or absence of epilepsy, whether participants have received training related to epilepsy, knowledge about epilepsy, and attitudes toward epilepsy.

Despite its enormous impact, to the best of our knowledge, there are no published studies on first aid practices of the public in the management of epileptic seizures and their associated factors in the south Gondar zone, Amhara region as a whole. Therefore, this study adds to the body of knowledge about epileptic seizure first aid practices and their associated factors. Moreover, assessing first aid practices among public in the management of epileptic seizures would hopefully serve as a stepping stone for future large-scale community-based studies, and would be used as an indicator to health managers for appropriate planning and intervention programs.

Thus, this study aimed to assess epileptic seizure first aid practices among public and their associated factors in Northwest Ethiopia in 2021.

## Materials and methods

### Study settings and period

A community-based cross-sectional study was conducted in the southern Gondar zone from February to April 2021. Based on the 2007 Census conducted by the Central Statistical Agency of Ethiopia (CSA), the southern Gondar zone has 468,238 households and a total population of 2,051,738. Of these, 1,041,061 are men and 1,010,677 are women. Debre Tabor town, a city in the southern Gondar zone, is located 666 km north of the capital city Addis Ababa. This zone has 21 districts and 406 subdistricts (Kebeles). There are 97 health centers and 10 hospitals in the community. Healthcare services for epilepsy and mental health issues exist in every hospital.

### Sample size determination and sampling procedures

In this study, the sample size was determined by using the single population proportion formula taking assumptions of a 95% confidence interval (CI), a 5% margin of error, and the magnitude of unsafe practices (66.5%) from a study conducted in the Oromia regional state, Ethiopia. The final sample size was 756 after adding 10% of the non-response rate to the sample size.

A multistage cluster sampling technique was used. First, the author randomly selects five districts from the total of 21 districts (Woreda) and three subdistricts (Kebeles) for each selected district. The source population include all households in the study area. All households found in the Kebeles were considered as the study population. Each person in the selected households for which actual data were collected was considered as the study unit. All people who lived permanently (at least 6 months) and were 18 years or older in the 20 selected Kebeles were included, excluding those who were seriously ill at the time of data collection.

To establish a sampling frame, a household survey was conducted by 40 health extension worker (HEW) data collectors 8 days before the actual data collection, and the household numbering was done in the selected Kebeles. The survey found a total of 8,789 people who ever helped people with seizures. Next, proportional allocation of the sample was made to identify representative samples from each Kebele based on the number of individuals who ever helped people with seizures. Then, a simple random sample technique (computer-generated random numbers) was employed. In situations there was more than one eligible study participant in a household, a lottery method was used to select only one.

### Study variables

The dependent variable was the community practice of first aid for patients with epilepsy during seizure episodes (unsafe/safe). Sociodemographic factors (like age, sex, place of residence, marital status, educational status, and employment status) the presence of members with epilepsy in a family, the presence of absence of epilepsy, participation or not in epilepsy-related training, knowledge about epilepsy, and their attitude toward epilepsy were independent variables.

### Data collection tools and procedures

Data were collected *via* face-to-face interviews using the previously adapted standard questionnaire that had two subsections. The first part dealt with the factors associated with the practice of seizure first aid measures. These factors included sociodemographic characteristics, familiarity with epilepsy, participation or not in epilepsy-related training, community knowledge about epilepsy, and their attitude toward epilepsy. The questions that measured the knowledge of the respondents about epilepsy had a sensitivity and specificity of 100 and 72%, respectively, and the entered data reliability was 0.87 (Cronbach's α). The attitude of the community toward epilepsy was measured using a six-point Likert scale questionnaire with an internal consistency value (Cronbach's α 0.79) (1 = I disagree very much, 2 = I disagree pretty much, 3 = I disagree a little, 4 = I agree a little, 5 = I agree pretty much, and 6 = I agree very much, that an item for which a “disagree” response (scored negatively has been reversed) indicates a positive attitude (38). In the second part, seizure first aid practice measures were categorized as safe/unsafe practices toward practice measures. The questionnaire was translated into Amharic by a bilingual translator. It was retranslated to the original version (English) to ensure consistency. Two HEWs as data collectors and two BSc nurse supervisors were selected for each selected Kebele. Data collectors and supervisors were trained by a principal investigator for 1 day, about the methods of data collection, its tools, and how to handle ethical issues. A pretest was conducted on 38 (5%) of the samples outside the study area before the actual data collection to find any potential problems with the data collection tools. Data collection was regularly monitored by a principal investigator and supervisors. Every day the collected data were checked for completeness and consistency before being promptly entered from paper to computer.

### Operational definitions

#### Practice

A 10-item questionnaire with yes/no options was used to assess the first aid practices of the community. Practice questions were graded with 1 point for each correct response and 0 point for each incorrect response. The total score is between 0 and 10. In this context, “safe practice” was defined as having a score greater than or equal to 50% on true questions about first aid practice measures, and an “unsafe practice” was defined as having a score <50% (19).

#### Training

Taking training was defined as whether the respondents took formal training or in any form of health education in health campaigns about the general information related to epilepsy, which has yes/no choices.

#### Knowledge

The knowledge of participants about epilepsy was assessed using a 10-item questionnaire with yes/no options. One point was awarded for each correct response to the true knowledge questions, while 0 point was awarded for each incorrect response. The total score is between 0 and 10. In this regard, good knowledge was defined when the respondents scored ≥50% in the true questions related to the knowledge about epilepsy, and “poor knowledge” was defined when respondents scored <50% on true knowledge questions (19).

#### Attitude

The part on attitude comprised 20 questions and response options on a six-point Likert scale consisting of I disagree very much (1), I disagree pretty much (2), I disagree a little (3), I agree a little (4), I agree pretty much (5), and I agree very much (6), that the items for which a “disagree” response (scored negatively have been reversed) indicates a positive attitude. Means were used as measures of the respondent's attitude. Participants whose score is equal to or greater than the mean have been considered to have a positive attitude whereas those whose score is less than the mean have been considered to have a negative attitude toward epilepsy related to the true attitude questions (38).

### Data processing and analysis

Data were coded, entered into Epi data version 4.2, and then exported to Statistical Package for Social Science (SPSS) version 24. To determine the association of independent variables with outcome variables, bivariable and multivariable binary logistic regression analyses were performed. Variables with *p* < 0.05 in bivariable analysis were taken to multivariable analysis for further analysis to control for confounding factors. The results were presented using frequencies, proportions, and odds ratio (OR) with a 95% CI, and variables with *p* < 0.05 were declared to be significantly associated with unsafe practices.

Model fitness was examined using Hosmer and Lemeshow's test (*p* = 0.45). Using the variance inflation factor and tolerance, multicollinearity was checked to determine the correlation between the independent variables. The variance inflation factor in this instance was 10, and the tolerance level was higher than 0.1, indicating that there was no dependence between the independent variables.

### Ethical considerations

Ethical clearance was obtained from the ethical review committee of Debre Tabor University, to obtain a permission letter from the south Gondar zone health department. The confidentiality of respondents was maintained using the anonymous data collection tool, and the questionnaire was provided with written consent. Selected personnel were informed that they can quit at any time, even if they had agreed to participate at first, and that their decision was not causing them any problems.

## Results

### Sociodemographic characteristics

A total of 741 respondents participated in this study with a response rate of 98.02%. Approximately half of the respondents were women 376 (50.7%) and urban residents 457 (61.7%). The mean age of the respondents was 30.1 years with a standard deviation (SD) of ± 12.5 years, with the majority of participants 534 (70.1%) included in the age group of 18–35 years. Most of participants were orthodox followers 610 (82.3%) and Amhara by ethnicity 711 (96.0%). Regarding their educational status, ~168 (22.7%) of them did not know how to write and read (Table 1).

**Table 1:** Sociodemographic characteristics of the community living in south Gondar zone, Amhara, Ethiopia 2021 (*n* = 741).

**Characteristics**	**Category**	**Frequency**	**Percent**
Sex	Male	365	49.3
	Female	376	50.7
Age	18–35	534	72.1
	36–45	122	16.4
	≥46	85	11.5
Residence	Rural	284	38.3
	Urban	457	61.7
Ethnicity	Amhara	711	96
	Oromia	20	2.7
	Tigray	10	1.3
Religion	Orthodox	610	82.3
	Muslim	94	12.8
	Catholic	10	1.3
	protestant	12	1.6
	Adventist	15	2.0
Educational status	Illiterate	168	22.7
	Primary school (1–8th grade)	138	18.6
	Secondary school (9–12th grade)	160	21.6
	College and above	275	37.1

### Familiarity, training, knowledge, and attitude of the community about epilepsy

In this study, 18 (2.4%) of participants had families with epilepsy, and nine (1.2%) of the respondents were living with epilepsy. Approximately 137 (18.5%) of them received epilepsy training, 59.9% of participants had poor knowledge about epilepsy, and 40.1% of participants had a negative attitude toward epilepsy.

## Seizure first aid practice of the community

In this study, the first aid practice of the community in managing people with episodes of epileptic seizures has been investigated. Of all participants, 641 (86.5%) take the patients to the holy water, 540 (72.9%) take the patient to prayer, 286 (38.6%) of the community took to the traditional healer, and 650 (87.7%) of the respondents refused to take them to the hospital. Similarly, 273 (36.8%) of participants insert clothing into the patient's mouth, and 318 (42.9%) of them restrain the patient from movement because they believed that this will reduce the seizure intensity. Approximately 460 (62.1%) of participants smoke the match in the belief that it treats epilepsy, 465 (62.8%) of them provide food and water to the patient while having a seizure episode, 185 (25.0%) of the respondents keep the patient away from harmful or sharp objects, and ~438 (59.1%) of them sprinkle water on the patient's body and believed that it is always the best treatment.

Overall, 71.7% (95%CI: 68.3, 74.9) of the south Gondar community had unsafe practice measures in managing patients with seizure episodes.

### Factors associated with seizure first aid practice

Bivariable and multivariable binary logistic regression analysis were conducted to show the relationship between independent variables and first aid practices. In the bivariable analysis, respondents who were rural residents, participants who had poor knowledge about epilepsy and a negative attitude toward epilepsy, respondents who did not take any training related to epilepsy, and participants who were illiterate were significantly associated with unsafe practice measures at *p* < 0.05. In contrast, participants who were young and reached secondary education were associated with safe practices. These variables were taken to multivariable analysis to control for confounding effects. In multivariable analysis, respondents who were illiterate, who had poor knowledge about epilepsy and a negative attitude toward epilepsy, as well as respondents who did not take any training related to epilepsy affected practice measures negatively. Conversely, participants who reached secondary education had safe practices in managing patients with seizure episodes.

When controlling for other variables, the odds of having unsafe practices among participants were 1.99 times higher among those who were illiterate compared to those who were in college and above [adjusted odd ratio (AOR) = 1.99, 95%CI: 1.00, 3.97] whereas individuals who reached secondary education had safe practice (AOR = 0.4, 95%CI: 0.26, 0.63). Similarly, participants who did not take training related to epilepsy had unsafe practices while managing patients on seizure episodes compared to those respondents who took training about epilepsy (AOR = 2.07, 95%CI: 1.35, 3.17). The likelihood of having an unsafe practice in managing seizures was greater among participants who had poor knowledge (AOR = 1.51, 95%CI: 1.06, 2.14) and a negative attitude (AOR = 2.20, 95%CI: 1.50, 3.22) compared to respondents who had good knowledge and a positive attitude toward epilepsy, respectively (Table 2).

**Table 2:** Factors associated with public first aid practice measures of an epileptic seizure, Northwest Ethiopia, 2021 (*n* = 741).

**Characteristics**	**Category**	**Practice**		**COR (95%CI)**	**AOR (95%CI)**	***P* value**
		**Safe**	**Unsafe**			
Age	18–35	190	344	*0.24 (0.12, 0.48)	0.48 (0.2, 1.15)	0.100
	36–45	10	112	1.49 (0.59, 3.76)	1.99 (0.72, 5.52)	0.186
	≥46	10	75	1	1	
Residents	Rural	60	224	*1.82 (1.29, 2.58)	0.78 (0.50, 1.22)	0.269
	Urban	150	307	**1**	1	
Educational status	Illiterate	19	149	*2.68 (1.55, 4.64)	**1.99 (1.00.3.97)	0.049
	Primary school	39	99	0.87 (0.55, 1.37)	0.72 (0.44, 1.19)	0.202
	Secondary school	82	78	*0.33 (0.22, 0.49)	*0.41 (0.26, 0.63)	0.000
	College and above	70	205	1	**1**	
Training	Yes	60	77	1	1	0.001
	No	150	454	*2.36 (1.61, 3.47)	**2.07 (1.35, 3.17)	
Knowledge	Good	109	188	**1**	**1**	0.024
	Poor	101	343	*1.97 (1.42, 2.72)	**1.51 (1.06, 2.14)	
Attitude	Positive	145	299	1	1	0.000
	Negative	65	232	*1.73 (1.23, 2.43)	**2.20 (1.50, 3.22)	

^*^Factors were significantly associated with pubic first aid practices of an epileptic seizure in bivariable analysis.

^**^Factors were significantly associated with pubic first aid practices of an epileptic seizure in multivariable analysis.

COR, crude odd ratio; AOR, adjusted odd ratio.

## Discussion

This study is based on the result of a previous research work, which showed that a greater number of communities in the northwest Ethiopia had poor knowledge and a negative attitude toward epilepsy (39). This study aimed to assess the first aid practice measures taken by the community in managing people with epileptic seizure episodes. This has been investigated in relation to sociodemographic variables and other factors that potentially affect their level of practice.

The magnitude of unsafe practices in the management of epileptic seizures in the south Gondar community was 71.7% (95%CI: 68.3, 74.9). Individuals who were illiterate and participants who did not take training related to epilepsy and had a poor knowledge about (AOR = 1.51, 95%CI: 1.06, 2.14) and a negative attitude toward epilepsy (AOR = 2.20, 95% CI: 1.50, 3.22) showed unsafe practices compared to their counterparts. Conversely, participants who reached secondary education had safe practice measures in the management of epileptic seizures.

In this study, 86.5% of participants took patients to holy water, 72.9% of them took patients to prayer, and 38.6% of the communities took patients to the traditional healer. Surprisingly, 87.7% of respondents refused to be taken to the hospital. This mismanagement is worse than that in the studies conducted in Mekelle, Cameron, India, and Nigeria. In a study conducted in Mekelle, 70.3% of respondents preferred to be taken to the holy water, 64.01% preferred to be taken to the hospital, 44.8% recommended to be taken to traditional healers, and 32.1% preferred to be taken to prayer (40). In a study conducted in Cameron, ~67.4% was taken to the hospital and 22.0% recommended prayers (41). In a study conducted in India, ~74.0% of participants calls a doctor in response to an epileptic seizure (42). Another research in Cameron found that 65.7% of them were taken to the doctor and 29.7% of participants were taken to prayer, and only 8.6% were taken to a traditional healer (43). In a study in Nigeria, the majority (87.4%) of participants agreed with hospital management of PWE (31). This difference might be due the differences in cultural background of participants. Moreover, the study in Mekelle was conducted among secondary students, while the current study was conducted on the general population. This calls the need for community education related to epilepsy. Similarly, in the Sudanese study, around 86.2% (44) of participants recommended medical treatment that was practiced safely compared to the current study. Here, the study was conducted in both rural and urban societies, while in the Sudanese study included only the residents of Khartoum. This suggests the need to disseminate information about epilepsy to the community as a whole, especially rural communities. Likewise, the Nigerian study was conducted among medical students. This enabled them to get the information through their course, and revealed the necessity for community health education on epilepsy. In this study, ~62.1% of participants smoke match sticks, which is a relatively safe practice compared to the Mekelle (81.9%) (40) study. Moreover, 36.8% of participants dangerously put clothes in patients' mouth, which seems to be better than the studies conducted in Cameron (41.6%) (43) and Nigeria (62.2%) (36) but worse than the studies conducted in Mekelle (22.8%) (40) and Addis Ababa (8.6%) (45). Approximately 318 (42.9%) of them restrain the patient from the movement as they believed that this will reduce the seizure intensity. This practice was relatively safe compared to studies in Nigeria (54.9%) (36) and India (47%) (42). This might be due to a variation in sociocultural background of the study participants. Moreover, 465 (62.8%) of them provide food and water to patients during a seizure episode. This was an unsafe practice compared to the studies in Mekelle (27.2%) (40) and Addis Ababa (41.7%) (45). Approximately 185 (25.0%) of the respondents move the patient away from harmful or sharp objects, which was a relatively unsafe practice compared to the studies conducted in Mekelle (59.0%) (40), Addis Ababa (58.3%) (45), Khartoum State Sudan (88.8%) (44), and India (61.0%) (42).

Overall, 71.7% (95%CI: 68.3, 74.9) of the south Gondar community had unsafe practice measures in managing patients with seizure episodes. This was unsafe practice compared to the studies in Jima Ethiopia (6.0%) (33), Lay Armachiho Ethiopia (55.3%) (34), Goncha Siso Enesie Woreda Ethiopia (63.2%) (35), and Nigeria (68.1%) (36). This discrepancy might be due to differences in study participants and cultural differences. In the Jima study, participants were epileptic, but in the current study all respondents were from the communities. In this case, PWE have a chance to receive continuous psychological education during their routine hospital visits, which helps them access safer practices compared to the general population. Similarly, in the current study, the community had unsafe practice measures compared to other studies conducted in Sululta Woreda Ethiopia (66.5%) (46) and Addis Ababa Ethiopia (59.1%) (45). This might be a difference in the deep-rooted sociocultural beliefs and practices about the causes and treatment of epilepsy. Moreover, in the current study, participants were from both urban and rural societies, whereas the Addis Ababa study included only teachers.

In relation to the factors that affect first aid practice, participants without epilepsy training and/or health education showed unsafe practices compared to those who did. This was in agreement with studies conducted in Nigeria (36), Addis Ababa (45), Lay Armachiho Ethiopia (34), and Sululta Woreda Ethiopia (46). In other words, training and/or health education will enable society to have better knowledge, which in turn helps to safely manage people in seizure episodes. Participants who were illiterate had unsafe practices and those who reached secondary education had safe practices in managing patients with seizure episodes, which were in line with a previous study conducted in Lay Armachiho Ethiopia (34). This might be that people who are illiterate did not have access to the information from different reading materials and did not gain knowledge that actually their level of practice become unsafe. Moreover, participants who had poor knowledge about epilepsy and negative attitudes toward epilepsy showed unsafe practices in the management of epileptic seizures. This finding was in agreement with the study done in Goncha Siso Enesie Woreda Ethiopia (35). It is theoretically agreed that people who had no information, i.e., poor knowledge and a negative attitude, will have unsafe practices compared with their counterparts.

## Conclusion

The findings of this study indicated that the majority of people in the south Gondar zone community have unsafe practices in the management of epileptic seizures. Factors such as illiteracy, lack of epilepsy-related training, and poor knowledge of and a negative attitude toward epilepsy were significantly associated with unsafe practices in first aid management of epileptic seizures.

This requires an emphasis on participants without epilepsy-related training and/or health education, those who are illiterate, and those with poor knowledge about and a negative attitude toward epilepsy. Moreover, this result revealed that there is still a gap in community health education that needs the direct engagement of health institutions to promote the awareness of communities about epilepsy and improve their attitude and practice. It is recommended that further qualitative studies, such as interviews with key informants (priests, traditional healers, etc.), are needed to explore the thoughts and motivations of religious and traditional healers on how to practice first aid in the management of epileptic seizures.

## Data availability statement

The raw data supporting the conclusions of this article will be made available by the authors, without undue reservation.

## Ethics statement

Ethical approval was obtained from IRB of Debre Tabor University. The patients/participants provided their written informed consent to participate in this study.

## Author contributions

All authors listed have made a substantial, direct, and intellectual contribution to the work and approved it for publication.

## Conflict of interest

The authors declare that the research was conducted in the absence of any commercial or financial relationships that could be construed as a potential conflict of interest.

## Publisher's note

All claims expressed in this article are solely those of the authors and do not necessarily represent those of their affiliated organizations, or those of the publisher, the editors and the reviewers. Any product that may be evaluated in this article, or claim that may be made by its manufacturer, is not guaranteed or endorsed by the publisher.

## Abbreviations

PWE: People with epilepsy
IRB: Institutional review board
SPSS: Statistical Package for Social Sciences
US: United States
WHO: World Health Organizations.
