# Supplementary material for: Epileptic seizure first aid practices of publics in Northwest Ethiopia 2021: Unsafe practices of nearly three-fourths of the community
Source: Front Neurol. 2022 Nov 15;13:1032479. doi: 10.3389/fneur.2022.1032479 (PMC9705738; doi:10.3389/fneur.2022.1032479)
Supplement: Supplementary file 1 [file Data_Sheet_1.pdf]

1. Annex

1.1. Consent form

Code No: _________

Good day;

My name is_________________; I am a data collector for the research which is conducted by Sintayehu Asnakew, and His research Colleagues who are lecturers at DTU. The purpose of the research is to assess Public first aid practices in managing epileptic seizure and its associated factors. This will be critical input for policy makers and health managers. Your participation in this research must be voluntary. If you decide not to participate; there will be no negative consequences on you. If you decide to participate there will be no benefits for you. However your participation on this study is very important for achievement of the study and for paving the way for development of intervention strategies in these areas. All the responses given by you; will be kept confidential using password. You are not expected to give your name or phone number. Without permission from you and legal body any part of this study will not be disclosed to third person. You have full right to refuse and withdrawal to participate in this study even if in the middle of the interview; if you don't wish. The interview period will take approximately about 20 minutes.

Are you voluntary to participate in the interview? 1. Yes 2.No

Name of contact person: Sintayehu Asnakew

E-mail- sintie579@gmail.com, Tel: +251918162236

Questionnaire

1. Socio-demographic characteristics of the participants

S. N	Characteristics	Response
1	Age in years	---
2	Sex	1. Male<br>2. Female
3	Residence	1. Rural.<br>2. Urban
4	Ethnicity	1. Amhara<br>2. Oromo<br>3. Tigray<br>4. Other ....
5	Religion	1. Orthodox<br>2. Muslim<br>3. Catholic<br>4. Protestant<br>5. Adventist<br>6. Others ----------
6	Educational status	1. Unable to read and write<br>2. Primary school<br>3. High school<br>4. College and above
7	Job status	1. Had job<br>2. Had no job

2. Training about and Familiarity with epilepsy

Sn.		Reponses	
		1. Yes	2. No
8	Had a family member with epilepsy		
9	Living with epilepsy		
10	Have you ever taken training about epilepsy?		

3. Questions for measuring Knowledge of the participants

S. N	Characteristics	Response	
		1. Yes	2. No
1	Epilepsy is a mental disease		
2	Epilepsy is uncontrollable disease		
3	Epilepsy is a hereditary disease.		
4	Epilepsy is a contagious through close contact with the patient		
5	All persons who convulses are epileptic		
6	Epilepsy is caused by ancestor's sin.		
7	Bad thoughts and evil spirits can cause epilepsy		
8	Being hit on the head, brain infections, high fever, problems with child delivery (labor) can cause epilepsy		
9	The symptoms of epilepsy during the attack are loss of consciousness, urine incontinence, trouble remembering after the attack , salivation and drooling, biting of the tongue , and up rolling of the eyes		
10	Some epileptic patients have cues about their attack		

4. Questions used to measure the attitude the participants

S.No	Characteristics	Response					
		1. disagree very much,	2. disagree pretty much	3. disagree a little	4. agree a little	5. agree pretty much	6. Agree very much
1	Schools should not place children with epilepsy into regular classrooms						
2	Persons with epilepsy have the same rights as all people						
3	Children with						

	epilepsy should attend schools for children with special needs						
4	The individual with epilepsy should not be prevented from having children						
5	Persons with epilepsy should be prohibited from driving						
6	Children with epilepsy should attend regular public schools						
7	Children need to be protected from classmates who have epilepsy						
8	Parents should expect of their child who has epilepsy what they expect of other children						
9	Persons with epilepsy should not be prohibited from marrying						
10	Persons with epilepsy prefer to live with others of similar characteristics						
11	Equal employment opportunities						

	should be available to individuals with epilepsy						
12	Families of children with epilepsy should not be provided supportive social services						
13	When their seizures are controlled by medication, persons with epilepsy are just like anyone else						
14	Persons with epilepsy can safely operate machinery						
15	Individuals with epilepsy are accident-prone						
16	Epilepsy is a degenerative condition that will lead to other mental problems						
17	Epilepsy and epilepsy medications can have a significant effect on students' mood, memory, and learning						

18	People with Epilepsy cannot lead a happy						
19	A PWE can be chief of the community						
20	Epilepsy is considered as a disability, therefore disability acts apply to it						

5. Questions used to measure first aid Practices for epileptic seizure

S. N.	Characteristics	Response	
		1. Yes	2. No
1	Taking to the Holly water is the best practice		
2	Taking to the hospital to be treated with antiepileptic medication		
3	Taking the a patient to the prayer		
4	Urgently taking to the traditional healer		
5	Providing food and water while the person is on seizure episode		
6	Inserting a cloth in the patients mouth		
7	Restraining the patient from movement during the attack decrease the seizure		
8	Smoking the match treats epilepsy		
9	Removing tight closes and harmful objects away from the patients		
10	Sprinkle water over the patient's body is always the best treatment		
